# Effect of Crosslinkers on Optical and Mechanical Behavior of Chiral Nematic Liquid Crystal Elastomers

**DOI:** 10.3390/molecules26206193

**Published:** 2021-10-14

**Authors:** Kyosun Ku, Kyohei Hisano, Kyoko Yuasa, Tomoki Shigeyama, Norihisa Akamatsu, Atsushi Shishido, Osamu Tsutsumi

**Affiliations:** 1 Department of Applied Chemistry, Ritsumeikan University, 1-1-1 Nojihigashi, Kusatsu 525-8577, Japan; gr0343xf@ed.ritsumei.ac.jp (K.K.); hisano@fc.ritsumei.ac.jp (K.H.); sc0065if@ed.ritsumei.ac.jp (K.Y.); sc0060kf@ed.ritsumei.ac.jp (T.S.); 2 Laboratory for Chemistry and Life Science, Tokyo Institute of Technology, 4259 Nagatsuta, Midori-ku, Yokohama 226-8503, Japan; akamatsu@res.titech.ac.jp (N.A.); ashishid@res.titech.ac.jp (A.S.)

**Keywords:** crosslinkers, chiral nematic liquid crystal elastomer, selective reflection, mechano-optical sensor

## Abstract

Chiral nematic (N*) liquid crystal elastomers (LCEs) are suitable for fabricating stimuli-responsive materials. As crosslinkers considerably affect the N*LCE network, we investigated the effects of crosslinking units on the physical properties of N*LCEs. The N*LCEs were synthesized with different types of crosslinkers, and the relationship between the N*LC polymeric system and the crosslinking unit was investigated. The N*LCEs emit color by selective reflection, in which the color changes in response to mechanical deformation. The LC-type crosslinker decreases the helical twisting power of the N*LCE by increasing the total molar ratio of the mesogenic compound. The N*LCE exhibits mechano-responsive color changes by coupling the N*LC orientation and the polymer network, where the N*LCEs exhibit different degrees of pitch variation depending on the crosslinker. Moreover, the LC-type crosslinker increases the Young’s modulus of N*LCEs, and the long methylene chains increase the breaking strain. An analysis of experimental results verified the effect of the crosslinkers, providing a design rationale for N*LCE materials in mechano-optical sensor applications.

## 1. Introduction

Chiral nematic (N*) liquid crystals (LCs) have attracted considerable scientific attention because of their fascinating optical properties that arise from the helical orientation of LC molecules in the nanoscale to microscale range [1,2,3]. The periodic helical structure of an N*LC spontaneously self-assembles when an achiral LC is mixed with chiral moieties and/or a chiral LC is used [4,5]. This helix has a refractive-index distribution along the helical axis and exhibits selective reflection of circularly polarized light with the same handedness at a specific wavelength according to Bragg reflection [6,7]. The peak reflection wavelength (*λ*_peak_) is related to the helical pitch (*P*) corresponding to the length of a 2π molecular rotation: *λ*_peak_ = *n*_ave_·*P*·sin *θ*, where *n*_ave_ is the average refractive index of ordinary (*n*_0_) and extraordinary (*n*_e_) refractive indices of the medium, and *θ* is the incident angle of the light [8,9]. Such optical properties enable the development of a wide range of N*LC-based optical applications in fields such as lasers [10], security inks [11], reflective displays [12,13], and sensors [14].

Crosslinked N*LC polymers are increasingly used for developing stimuli-sensitive devices [15]. Crosslinking the N*LC polymer network can stabilize the helical molecular orientation. Under external stimuli, the molecular orientation and corresponding material properties change, and when the stimuli are removed, the original molecular orientation is recovered [16,17,18]. Crosslinked N*LC polymer networks are classified by crosslinking density into densely crosslinked LC networks (LCNs) or loosely crosslinked LC elastomers (LCEs); in particular, the LCEs swelled by low-molecular-weight LCs are termed LC gels [19]. Due to the dense crosslinking, LCNs are environmentally stable, but their stimuli responsivity is quite low [20,21]. In contrast, LCEs show higher sensitivity and reversibility under external stimuli, which are favorable for stimuli-responsive applications [22,23]. In particular, the N*LCE enables selective reflection, where *λ*_peak_ changes under external stimuli. Thus, N*LCEs are key components for developing electro-, thermo-, or mechano-responsive optical sensors with excellent repeatability and sensitivity [24,25,26,27,28].

Such N*LCEs have been widely synthesized using various polymerization systems and chemical components [29,30,31,32]. Previous studies suggest that crosslinking units affect the physical properties of N*LCEs, including their phase transition behavior, thermal stability, optical properties, and mechanical properties. Once crosslinking units are optimized for desired optical properties, other materials’ properties may be deteriorated [33,34,35]. In particular, the relationship between crosslinkers in chemical systems and the resultant optical properties arising from the ability of chiral dopants to generate helical molecular orientations, i.e., the helical twisting power (HTP), has rarely been examined. Typically, HTP is expressed as follows: HTP = (*P*·*c*)^−1^, where *c* is the molar fraction of the chiral dopant [36]. Although HTP is important for designing the selective reflection color, it depends on the chemical system used, and the concentration of the active components must be optimized in each system. Therefore, a good understanding of the relationship between the crosslinker structure and HTP is necessary for providing us with a synthetic strategy for the creation of both N*LCEs and other materials with desired optical properties.

In this study, to investigate the effect of crosslinking units in N*LCE materials on both optical and mechanical properties, we synthesized N*LCEs with various crosslinkers: flexible methylene chains with different lengths and/or mesogenic units. The resultant N*LCEs showed mechano-responsive reflection color changes under applied tensile strain, indicating that the proposed system is applicable to mechano-optical sensors. Similar shifts in *λ*_peak_ under strain were observed, irrespective of whether the crosslinkers had mesogenic units. Furthermore, the thermal, optical, and physical properties of the N*LCEs were characterized. Increasing the length of the methylene chains had almost no effect on the thermal and optical properties of the resultant N*LCEs. In chemical systems using a non-mesogenic crosslinker, the HTP of the chiral agent was consistent with previously reported values [37]; in particular, the system with a mesogenic crosslinker had a smaller HTP value than those without mesogenic units, meaning that the optical property differed from the expected one. Our results revealed that the low HTP value in the mesogenic crosslinker system is due to the larger molar ratio of mesogenic compounds (the sum of the LC monomer and mesogenic crosslinker). Thus, by setting the molar ratio of the chiral agent to that of all mesogenic compounds, the HTP became equal to that of the non-mesogenic crosslinking system. This strategy enables mechano-optical sensors to be designed with a desired initial color by tailoring the relative amounts of chiral agents, while the other material properties can be tuned by changing the crosslinkers.

## 2. Results and Discussion

### 2.1. Phase Transition Behavior

**Scheme 1:**
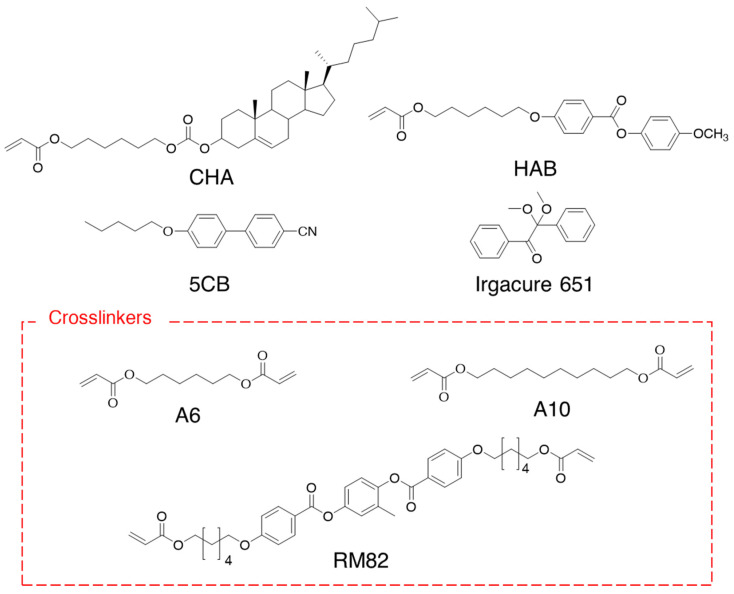
Chemical structures of the compounds used in the synthesis of N*LCEs.

**Figure 1:**
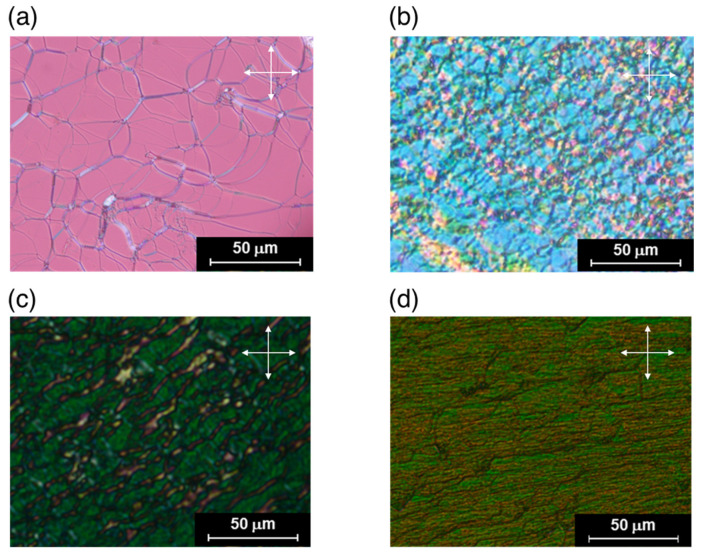
POM images of N*LC samples before and after photopolymerization: (**a**) E-A6 before photopolymerization (as a representative example); (**b**) E-A6 after photopolymerization; (**c**) E-A10 after photopolymerization; (**d**) E-RM82 after photopolymerization.

Structures of monomeric materials used for preparation of the N*LCEs are shown in Scheme 1. The phase transition temperatures of the monomer mixtures and N*LCEs are summarized in Table 1. As shown in Figure 1, polarized optical microscopy (POM) observations revealed that at the LC temperature, the monomer mixtures exhibit a Grandjean texture, which is typically observed for the homogenous helical molecular orientation in the N*LC phase. The N*LC-to-isotropic phase transition temperature (clearing point, *T*_c_) and crystalline-to-N*LC phase transition temperature (melting point, *T*_m_) of the monomer mixtures were slightly decreased by increasing the methylene chain length in the crosslinking unit. This demonstrates that increasing the methylene chain length in the crosslinking units contributes to destabilizing the LC phase by increasing the volume of non-LC units. Generally, non-LC-type molecules destabilize the LC phase by disturbing the formation of the LC molecular arrangement [38]. In contrast, *T*_c_ and *T*_m_ of the monomer mixture with a mesogenic crosslinker, RM82, were observed at 53 °C and 22 °C, respectively. These results indicate that RM82 contributes to the stabilization of the LC phase.

**Figure 2:**
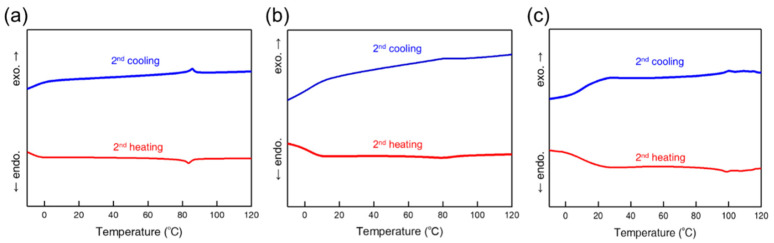
DSC thermograms of N*LCEs on the second scanning cycles from −10 °C to 120 °C: (**a**) E-A6, (**b**) E-A10, and (**c**) E-RM82. The scanning rate was 10 °C min^−1^.

The phase transition temperatures were determined by POM (Figure 1) for the monomer mixture and by differential scanning calorimetry (DSC) for N*LCE in the 2nd cooling process (Figure 2).

After photopolymerization of the mixtures, we obtained an N*LC polymeric film with a Grandjean texture, as shown in Figure 1. The films exhibited a reversible phase transition from N*LCs to isotropic phases with increasing temperature, and vise versa. The DSC curves indicated the glass transition temperature (*T*_g_) and *T*_c_ of the films, as shown in Figure 2. Although the *T*_g_ of the resultant films was slightly different depending on the chemical structure of the crosslinkers, they all showed a *T*_g_ below room temperature (~25 °C). Therefore, all the resultant polymers serve as elastomers at room temperature. Considering that typical acrylic polymers show a *T*_g_ of ~40 °C, the *T*_g_ of the reported N*LCEs is quite low. This is due to the use of 5CB, a low-molecular-weight, room-temperature LC, which swells the polymer network to serve as a plasticizer and activates the micro-Brownian motion. The *T*_c_ values of E-A6 and E-A10 were similar, 82 °C and 84 °C, respectively. E-RM82 showed a higher *T*_c_ than E-A6 or E-A10. This demonstrates that the π-conjugation of the aromatic ring in the RM82 unit increased *T*_c_ by increasing molecular order and rigidity [39,40]. Therefore, N*LCEs with a gel system can function as elastomers, irrespective of the chemical structure of the crosslinkers, where the stability of the N*LC phase can be increased using a mesogenic crosslinker. The results exhibit that the phase transition temperature is controlled by a crosslinker and provides high thermal stability in N*LCE. The high thermal stability of the N*LC phase extends the range of actuation temperature and allows various application fields in extreme environmental fields as a mechano-optical sensor.

### 2.2. Optical Properties of N*LCEs

**Figure 3:**
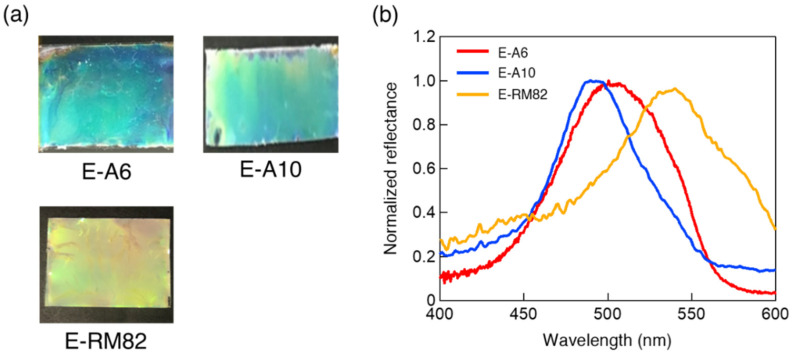
(**a**) Photographs and (**b**) normalized reflectance spectra of E-A6, E-A10, and E-RM82.

The E-A6 and E-A10 had a bright bluish-green color, while E-RM82 appeared orange (Figure 3a). Figure 3b shows the reflectance spectra of the N*LCEs; the *λ*_peak_ values were 493 nm (E-A6), 501 nm (E-A10), and 539 nm (E-RM82). As mentioned previously, the *λ*_peak_ of N*LCEs changes when mesogenic crosslinkers are used instead of methylene chains. According to the general knowledge of chiral transfer from chiral agents to achiral nematic LCs, the HTP of a chiral agent, as well as *λ*_peak_, should not change significantly with different crosslinkers [41,42]. However, the N*LCEs exhibited different *λ*_peak_ at the initial state, and the N*LCE with a mesogenic crosslinker (E-RM82) had a larger value (Figure 3b). To investigate the relationship between the chemical systems with mesogenic crosslinkers and the HTP of a chiral agent, we evaluated HTP by using HTP = (*P*·*c*)^−1^ and *λ*_peak_ = *n*_ave_·*P*, where *n*_ave_ was assumed to be 1.6 [43,44,45]. As a result, the HTP values of the N*LCEs were 10.8 µm^−1^ (E-A6), 10.6 µm^−1^ (E-A10), and 9.89 µm^−1^ (E-RM82). We assumed that the slight decrease in HTP which occurred only in the N*LCE with a mesogenic crosslinker (E-RM82) was due to the difference in the total molar ratio of the mesogenic compounds. Thus, we recalculated the HTP in the system of E-RM82 by using the molar ratio of chiral agent (=28 mol%) to the total mass of mesogenic compounds, which is the sum of concentrations of HAB, 5CB, and RM82. As a result, the recalculated HTP in the system of E-RM82 becomes 10.6 µm^−1^.

**Figure 4:**
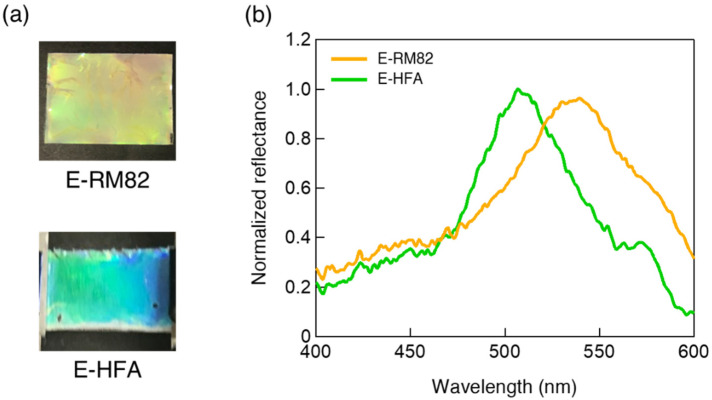
(**a**) Photographs and (**b**) normalized reflectance spectra of E-RM82 and E-HFA.

To further investigate this assumption, we prepared another N*LCE, referred to as E-HFA, with a mesogenic crosslinker, where the molar ratio of the chiral agent to the total amount of mesogenic compounds was set as 30 mol% (Table 2). As shown in Figure 4a, E-HFA had a bluish-green appearance, similar to E-A6 and E-A10. Furthermore, the *λ*_peak_ of E-HFA was 507 nm (Figure 4b), similar to that of E-A6 and E-A10. These results suggest that HTP is related to the molar ratio of not only a chiral agent in the chemical system, but also mesogenic compounds. This provides a strategy for designing mechano-optical sensors with the desired initial reflection color by adding certain amounts of chiral agents, irrespective of the crosslinker.

### 2.3. Mechano-Optical Properties

**Figure 5:**
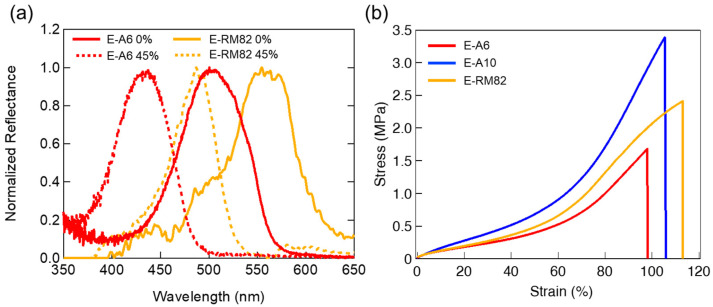
(**a**) Reflectance spectra of E-A6 and E-RM82 under tensile strain of 0% (solid line) and 45% (dashed line). (**b**) Stress–strain curves of the N*LCEs of E-A6, E-A10, and E-RM82. The strain was varied at a rate of 2 mm min^−1^.

We investigated the mechano-optical properties of N*LCEs by reflection spectroscopy using a UV-vis spectrometer, where the incident light is delivered through an optical fiber and is normally irradiated to the film. The reflected light was detected by a photodiode array detector at the same angle. As representative examples, we investigated the effect of crosslinkers on the mechano-optical properties using E-A6 and E-RM82. Figure 5a compares the reflectance spectra of E-A6 and E-RM82 under no strain and 45% strain. Elongation of both samples to 45% strain resulted in a blue-shift of the *λ*_peak_, irrespective of the initial color (to 431 nm for E-A6 and to 484 nm for E-RM82). Considering Bragg’s reflection formula with normal incident light, the change in the helical pitch can be expressed as follows: Δ*λ*_peak_ = *n*_ave_·Δ*P*. By transforming the formula, we evaluated the rate of change of the helical pitch: Δ*λ*_peak_/*λ*_peak_ = Δ*P*/*P*, giving values of 14% and 13% for E-A6 and E-RM82, respectively. This indicates that the presented N*LCEs exhibit the same mechano-optical behavior, even with different crosslinkers. Typical LCEs exhibit different deformation behaviors depending on the chemical structure of the crosslinker. In our system, N*LCEs can be regarded as a gel swelled by a low-molecular-weight LC; thus, the deformation behavior is similar to that of the non-crosslinked system, and the effect of crosslinkers on molecular reorientation behavior is negligible.

To further understand the effect of crosslinkers on the mechanical deformation behavior of N*LCEs, we conducted a tensile test, as shown in Figure 5b. The Young’s modulus and breaking strain of each film were 2.1 MPa and 98% strain (E-A6), 2.0 MPa and 105% strain (E-A10), and 2.7 MPa and 113% strain (E-RM82), respectively. The methylene chain length of the crosslinkers had no effect on the Young’s modulus, but the N*LCE with a mesogenic crosslinker had higher stiffness. This can be explained by considering that aromatic π-conjugation has a stronger interaction than the methylene chains, which increases mechanical stiffness [46]. In addition, longer methylene chains in the crosslinker increased the breaking strain. This is reasonable because the N*LCEs have the same crosslinking density but different methylene chain lengths, in which the N*LCEs with longer methylene chain lengths deform over a wider range. These results provide the effect of a crosslinker in the mechano-optical actuation of N*LCE, indicating that mechanical properties such as stiffness and stretchability can be independently controlled by using a different crosslinker. Moreover, this strategy can be used to design a mechano-optical sensor that satisfies the mechanical requirements of practical applications.

## 3. Materials and Methods

### 3.1. Materials

The chemical structures of the materials used to prepare the N*LCEs are shown in Scheme 1. A LC monomer (HAB) and a mesogenic crosslinker (RM82) were supplied by Osaka Organic Chemical Ind. Ltd. (Osaka, Japan) and were used after recrystallization from methanol. Non-mesogenic crosslinkers (A6 and A10) were purchased from a commercial supplier and used after purification by silica gel column chromatography using dichloromethane. A low-molecular weight LC (5CB) and a photoinitiator (Irgacure 651) were purchased from commercial suppliers and used as received. A chiral agent (CHA) was synthesized and purified as described previously [28,47]. The polydimethylsiloxane film (removal layer) was prepared as previously reported [27].

### 3.2. Synthesis of CHA

6-(carboxyoxy)hexyl acrylate (1.0 g, 6.0 mmol) and pyridine (0.47 g, 6.0 mmol) were added to dry dicholro methane (CH_2_Cl_2_, 50 mL) and stirred for 5 min at 0 °C. Cholesteryl chloroformate (2.7 g, 6.0 mmol) was added to the mixture and stirred for 19 h at r.t. CH_2_Cl_2_ was then added to the reaction mixture and washed with distilled water and a saturated sodium chloride (NaCl) aqueous solution. The organic layer was dried with anhydrous sodium sulfate (Na_2_SO_4_). The crude product obtained was purified by silica gel column chromatography (CH_2_Cl_2_) and recrystallization (methanol) to obtain CHA. Yield: 48% as needle-like crystal ^1^H NMR (400 MHz, CDCl_3_, *δ*): 6.39 (dd, *J* = 17.3, 1.5 Hz; 1H; CH*H*=CH), 6.10 (dd, *J* = 17.3, 10.3 Hz; 1H; CH_2_=C*H*), 5.81 (dd, *J* = 10.3, 1.5 Hz; 1H; C*H*H=CH), 5.38 (d, *J* = 5.2 Hz; 1H; in steroid), 4.49–4.41 (m, 1H; in steroid), 4.15–4.04 (m, 4H; CH_2_=CHCOOC*H*_2_(CH_2_)_4_C*H*_2_), 2.42–2.32 (m, 2H; in steroid), 2.01–1.76 (m, 5H; CH_2_=CHCOOCH_2_C*H*_2_(CH_2_)_2_C*H*_2_CH_2_ and steroid), 1.68–1.58 (m, 5H; in steroid), 1.57–0.80 (m, 36H; CH_2_=CHCOO(CH_2_)_2_(C*H*_2_)_2_(CH_2_)_2_) and steroid), and 0.65 (s, 3H; in steroid). ^13^C NMR (100 MHz, CDCl_3_, *δ*): 166.42, 154.73, 139.46, 130.71, 128.63, 123.02, 77.78, 67.71, 64.56, 56.75, 56.18, 50.05, 42.38, 39.78, 39.60, 38.12, 36.93, 36.62, 36.25, 35.88, 31.98, 31.90, 28.67, 28.58, 28.32, 28.11, 27.78, 25.72, 25.55, 25.36, 23.90, 22.94, 22.67, 21.12, 19.36, 18.80, and 11.95. MS (ESI+) *m*/*z* [M + Na]^+^ calcd. for C_37_H_60_NaO_5_ 607.4333; found 607.4352. Anal. Calcd. for C_37_H_60_O_5_: C, 75.98; H, 10.34; O, 13.68. Found: C, 75.81; H, 10.71; N, 0.14.

### 3.3. Preparation of Polydimethylsiloxane Films

Glass plates (70 × 50 mm^2^) were dipped in a bath of 2.0 g L^−^^1^ ethanol solution of octadecyltriethoxysilane for 30 min at 60 °C. After the glass plates were heated at 120 °C for 2 h, a pair of glass plates was assembled to prepare polymerization cells, where the gap between the glass plates was adjusted to 110 µm with Kapton^®^-tape spacers. The precursor of polydimethylsiloxane elastomer with 10 wt% of a curing agent (Dow Corning Toray, Sylgard 184) was injected into the cell at room temperature. After the cell was cured at 75 °C for 3 h, a polydimethylsiloxane film with 110 µm thickness was removed from the cell.

### 3.4. Preparation of N*LCEs

**Figure 6:**
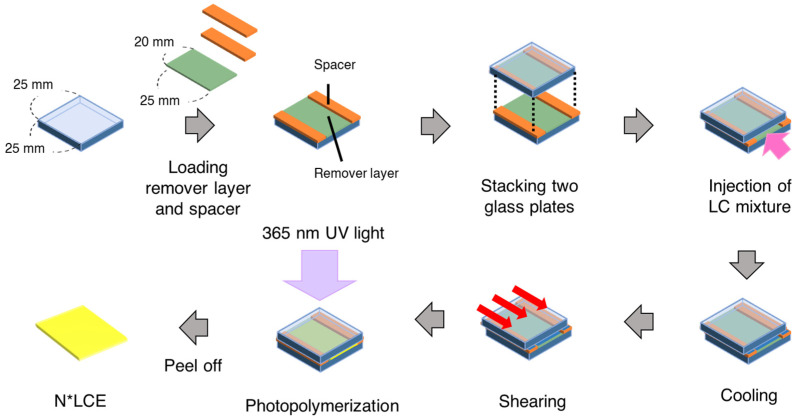
Schematic of the fabrication process for synthesizing N*LCEs.

Three types of N*LCEs were prepared using different crosslinkers. To prepare the monomer mixtures, HAB, CHA, 5CB (as a plasticizer), Irgacure 651, and the crosslinker were dissolved in tetrahydrofuran (THF); the solvent was then removed completely under reduced pressure. The molar ratios of the compounds in the monomer mixtures are summarized in Table 3. The sum of LC monomers and plasticizer was set to 100 mol%, and 7 mol% of crosslinker and 1 mol% of photoinitiator were used to prepare the N*LCE. The synthesis procedure for the N*LCEs is schematically illustrated in Figure 6. A polydimethylsiloxane film (removal layer, 20 × 25 mm^2^, and 110 µm thickness) and Kapton^®^-tape spacers (110 or 165 µm thickness) and were adhered to a glass plate (25 × 25 mm^2^). A pair of these glass plates was then assembled to give a polymerization cell with a 55 µm gap between the removal layers. The monomer mixture was injected into the gap by capillary force at 80 °C (isotropic temperature of the mixture) in the dark, and then the cell was cooled to 25 °C (N*LC phase temperature of the mixture). A shearing force was applied parallel to the injection direction of the LC mixture to induce homogeneous alignment of the helical molecular orientation in the N*LC phase. The mixture was photopolymerized by irradiation with a UV light-emitting diode (Iwasaki Electric Co. (Tokyo, Japan), LHPUV365; 365 nm; 5.0 mW/cm^2^) for 5 min at 25 °C. Finally, N*LCEs (20 × 25 mm^2^ and 55 µm thickness) were peeled off the glass cell.

### 3.5. Characterization

Nuclear magnetic resonance (NMR) spectroscopy was performed on a spectrometer (JEOL (Tokyo, Japan), ECS400; 400 MHz for H-NMR, 100 MHz for C-NMR) in the NMR solvent as an internal reference. Mass spectroscopy measurements were recorded by electrospray ionization (ESI) technique using a Bruker MICROTOF II (Billerica, MA, USA) and elemental analysis was measured using a MICRO CORDER JM10 (J-SCIENCE, Tokyo, Japan), respectively. The thermodynamic properties of the N*LCEs were analyzed using DSC (X-DSC7000, SEIKO Instruments, Chiba, Japan). DSC scans were performed within −10~120 °C at a scanning rate of 10 °C min^−1^ under nitrogen. The phase sequences and phase transition behaviors were determined using a POM instrument (Olympus, Tokyo, Japan, EX51TH) equipped with a hot stage (Instec, Boulder, CO, USA, HCS302 hot stage, and mK1000 controller). The phase transition behavior and DSC measurement were repeated for 3 cycles and the same results investigated regardless of cycle. The reflectance spectra were recorded using a UV-vis diode-array spectrometer (BLUE-WaveUVN, Stellar Net, Tampa, FL, USA) at room temperature at a tensile strain of 0% and 45% using digital venire calipers. Stress–strain curves were recorded using a tensile tester (Instron 5943, Instron) at room temperature with a strain rate of 2 mm min^−1^. Three specimens were prepared for evaluating the Young’s modulus of N*LCEs and the values were averaged. The Young’s modulus of the N*LCEs was determined from the slope of the stress–strain curve in the linear elastic range of 0.25–1.0%. The test specimens of the N*LCEs with the size of 20 × 5 mm^2^ × 55 µm were prepared by manually cutting the bare N*LCEs with the size of 20 × 25 mm^2^ × 55 µm.

## 4. Conclusions

To investigate the effect of the crosslinking units in a N*LCE network, N*LCEs were synthesized with various crosslinkers, and the phase transition behavior as well as thermal, optical, and mechanical properties were investigated. The N*LCEs exhibited a bright color due to selective reflection and a reversible thermal phase transition. Long methylene chain units on the crosslinker increased the *T*_g_ and *T*_c_ of the N*LCEs. The LC-type crosslinker contributed to stabilizing the N*LC phase by increasing molecular order and rigidity. The LC-type crosslinker decreased the HTP of N*LCEs by increasing the molar ratio of the mesogenic compounds. The N*LCEs showed the same optical shift trend by mechanical deformation despite the presence of different types of crosslinker, accompanied by variations in the magnitude of the blue-shift in the reflectance. Moreover, the mechanical properties of N*LCE can be controlled using crosslinkers. These findings provide guidelines for the synthesis of improved N*LCE materials with enhanced performance as stimuli-responsive materials.

## Figures and Tables

**Table 1:** Phase transition temperatures of monomer mixtures and N*LCEs with different crosslinkers.

Crosslinker	Phase Transition Temperature *^a^* (°C)	
	Monomer Mixture	N*LCE
A6	C 22 N* 31 I	G 0 N* 82 I
A10	C 12 N* 26 I	G 2 N* 84 I
RM82	C 22 N* 53 I	G 11 N* 100 I

*^a^* Phase transition temperature of compounds; C, crystal; G, glass; N*, chiral nematic; I, isotropic.

**Table 2:** Molar ratios of the compounds used to synthesize E-RM82 and E-HFA.

N*LCE	Monomer	Chiral Agent	Plasticizer	Crosslinker	Photoinitiator
	HAB	CHA	5CB	RM82	Irgacure651
E-RM82	50	30	20	7.0	1.0
E-HFA	45	30	18	6.4	1.0

**Table 3:** Molar ratio of the compounds in the monomer mixtures used to prepare the N*LCEs.

N*LCE	Monomer	Chiral Agent	Plasticizer	Crosslinker			Photoinitiator
	HAB	CHA	5CB	A6	A10	RM82	Irgacure 651
E-A6	50	30	20	7.0	-	-	1.0
E-A10	50	30	20	-	7.0	-	1.0
E-RM82	50	30	20	-	-	7.0	1.0

## Data Availability

The authors confirm that the data supporting the findings of this study are available within the article.

## Sample Availability

Samples of the compounds are not available from the authors.
